# *Lactiplantibacillus pentosus* P2020 protects the hyperuricemia and renal inflammation in mice

**DOI:** 10.3389/fnut.2023.1094483

**Published:** 2023-02-20

**Authors:** Zhihuan Wang, Liqiong Song, Xianping Li, Yuchun Xiao, Yuanming Huang, Yue Zhang, Jintong Li, Mingding Li, Zhihong Ren

**Affiliations:** ^1^State Key Laboratory for Infectious Disease Prevention and Control, National Institute for Communicable Disease Control and Prevention, Chinese Center for Disease Control and Prevention, Research Units of Discovery of Unknown Bacteria and Function (2018 RU010), Chinese Academy of Medical Sciences, Beijing, China; ^2^Third Affiliated Hospital, Beijing University of Chinese Medicine, Beijing, China; ^3^Dongzhimen Hospital, Beijing University of Chinese Medicine, Beijing, China; ^4^Maiyata Institute for Beneficial Bacteria, Shaoxing, Zhejiang, China

**Keywords:** hyperuricemia, probiotics, *Lactiplantibacillus pentosus*, gut microbiota, kidney inflammation, urate reabsorption transporter, NF-kB, intestine barrier function

## Abstract

**Introduction:**

Hyperuricemia (HUA) is a common metabolic disease, and its prevalence has been increasing worldwide. Pharmaceutical drugs have been used for controlling HUA but they all have certain side effects, which thus calls for discovering alternative options including using treatment of probiotics to prevent the development of HUA.

**Methods:**

We established HUA mice model induced by potassium oxonate and adenine and performed in vivo experiments to verify the ability to lower serum uric acid of *Lactiplantibacillus pentosus* P2020 (LPP), a probiotics stain extracted from Chinese pickle. We also tried to discussed the underlying mechanisms.

**Results:**

Oral administration with LPP significantly decreased serum uric acid and reduced renal inflammatory response by downregulating multiple inflammation pathways including NK-kB, MAPK, and TNFα. We also found that LPP administration significantly promoted uric acid excretion by regulating expression of transporters in the kidney and ileum. In addition, LPP intake improved intestinal barrier function and modulated the composition of gut microbiota.

**Discussion:**

These results suggest that probiotics LPP may have a promising potential to protect against development of HUA and HUA-related renal damage, and its working mechanisms involve regulation of inflammation pathways and expression of transporters in the kidney and ileum.

## 1. Introduction

Hyperuricemia (HUA), one of the common metabolic diseases, is caused by abnormal purine metabolism and/or decreased uric acid excretion. People with serum uric acid (SUA) levels higher than 420 μmol/l in men and 360 μmol/l in women can be diagnosed as HUA. Globally, the prevalence of HUA and gout both have increased over the last few decades. The prevalence of HUA in Chinese adults was 8.4%, accounting for approximately 92.9 million adults with HUA (1). The consequence of long-term HUA can lead to chronic kidney disease (CKD), known as hyperuricemic nephropathy (2). Moreover, asymptomatic HUA, which has drawn less attention from researchers and the public, is related to in the creased prevalence of multiple diseases including hypertension, acute and chronic kidney disease, metabolic syndrome, obesity, and diabetes mellitus fatty liver (3, 4). Several mechanisms responsible for HUA development and urate-induced renal inflammation have been described. The presence of urate crystal can promote inflammasome-independent mechanisms, including releasing pro-inflammatory cytokines and neutrophil extracellular trap inflammation. Urate crystal also has pro-oxidative effect in multiple cell types and it activates inflammatory signaling through multiple mechanisms including activation of MAPK pathway and AKT–mTOR, and inhibition of AMPK (5–7).

Several methods are currently available to control HUA, such as diet, sport, drugs, and biotherapy, aiming to suppress HUA production or increase uric acid excretion (8). For many patients with HUA, dietary therapy for reducing the intake of high-purine is a low-cost and side effect-free or low side effect strategy with poor patient compliance (9, 10). Allopurinol, the representative of the HUA-lowering drug, can compete with the xanthine oxidase (XOD) enzyme to inhibit production of uric acid. Uricosuric agents such as benzbromarone, sulphinpyrazone, and probenecid can promote excretion of uric acid. Although the safety of XOD inhibitors and uricosuric agents has been improved, their adverse effects, such as headaches, diarrhea, rashes, severe allergic reactions, and nephrotoxicity, often limit their clinical use (11, 12). Therefore, it is necessary to find an effective intervention with better patient compliance and fewer side effects.

Gut microbiota is associated with multiple diseases and also plays an important role in the metabolism of purine and uric acid. Gout patients had a different gut microbiome pattern characterized by a significant decrease in bacteria expressing the uricase gene. In addition, the altered gut microbiota influences expression of UA transporters (e.g., ABCG2 and GLUT9) in the intestine and the systematic inflammation reaction, both of which contribute to the development of HUA (6, 13, 14). Thus, it is possible that probiotics can be used to ameliorate HUA and relieve the inflammatory damage caused by urate in urine. Researchers have found that several strains of *Lactobacillus* can reduce the levels of serum uric acid. For example, *Limosilactobacillus fermentum* JL-3 isolated from the Chinese traditional food “Jiangshui” was capable of degrading uric acid *ex vivo* and alleviating HUA in the mouse model (15). *Lactobacillus gasseri* PA-3 can directly utilize the purine compounds including adenosine monophosphate (AMP), inosine monophosphate (IMP), and guanosine monophosphate (GMP) for growth, and decrease the purine absorption in rats (16, 17). In the present study we isolated a strain named *Lactiplantibacillus pentosus* P2020 (LPP) from the Chinese pickle and evaluated its anti-HUA function using a mouse model. Our results showed that administration of LPP significantly downgraded the inflammation in kidney. We also observed the altered expression of transporters related to urate transportation in both kidney and ileum. It has been reported that several probiotics can improve epithelial barrier function and rebuild the disrupted intestinal flora (15, 17, 18). We found that LPP treatment upregulated expression of tight junction protein and reversed the changes in gut microbiota induced by HUA. Therefore, LPP can be considered a potential probiotic candidate to lower HUA, which warrants it to be further evaluated in human trials.

## 2. Materials and methods

### 2.1. Ethics statement

This study was approved by the Ethics Review Committee of the National Institute for Communicable Disease Control and Prevention at the Chinese Center for Disease Control and Prevention (Beijing, China).

### 2.2. Bacteria strain culturing

LPP was isolated from a Chinese pickle. The strain was grown anaerobically in Man-Rogosa-Sharpe (MRS) medium in a CO_2_ incubator (Forma CO_2_, Thermo Fisher Scientific, Waltham, MA, United States) at 37°C for 24 h before use. The culture of LPP in the logarithmic phase was collected and washed twice with sterile phosphate-buffered saline (PBS) by centrifugation (2000xg, 10 min, 4°C) and then resuspended with sterile PBS for oral inoculation to mice.

### 2.3. *Lactiplantibacillus pentosus* P2020 strain identification based on the 16S rDNA sequence and genome sequence

The 16S rDNA sequence and genome sequence were used to identify LPP at the species level. The genomic DNA was extracted using FastPure DNA isolation mini kit (Vazyme Biotech Co., Ltd., Nanjing, China). The 16S rDNA sequence was amplified using two universal primers, 27F and 1492R, and the draft genome was sequenced by the Illumina HiSeq TM2000 platform.

The nearly complete 16S rDNA sequence (1,567 bp) was obtained and blasted using the Ezbiocloud database (ChunLab Inc., Seoul, Korea) to search for similar sequences. LPP had the highest similarity to *Lactiplantibacillus pentosus* DSM 20314 T (= ATCC 8041 T, NCDO 363 T, NCIB 8026 T) at 99.93%.

For further identification, genome comparative analysis was carried out. As a reference strain, the whole-genome sequence of *Lactiplantibacillus pentosus* DSM 20314 T (CP032757) was obtained from the NCBI GenBank database. The digital DNA–DNA hybridization (dDDH) was assessed using the DDH web software^1^. The average nucleotide identity (ANI) was estimated by an online ANI calculator^2^. The dDDH and ANI values of genome sequences between strain P2020 (3,876,975 bp) and *Lactiplantibacillus pentosus* DSM 20314 T (CP032757) were 98.00 and 99.74%, respectively, which were much higher than the 70.0% and 95.0–96.0% threshold of dividing into species (19, 20). Thus, LPP was considered to be a strain of *Lactiplantibacillus pentosus*.

### 2.4. Animal experiment

Male Kunming mice (6-week-old, 25 ± 2 g) were purchased from the Chinese Beijing Vital River Laboratory Animal Technology Co., Ltd. All mice were housed in the Animal Center of China CDC Mice with temperature at 24–26°C, humidity at 40–60%, and a 12 h light–dark cycle. Mice had free access to the standard commercial mouse food and water. After one-week adaption, mice were randomly divided into three groups (*n* = 8/group): negative control group (NC), hyperuricemic group (HUA), and LPP-treated group (LPP). The HUA mouse model was established according to literature with slight modification (18, 21–26). Briefly, potassium oxonate (Sigma-Aldrich, MA, United States,) and adenine (Sigma-Aldrich, MA, United States) were suspended in 0.5% Carboxymethylcellulose sodium (CMC-Na) solution. Mice in HUA and LPP groups were daily administered with 0.3 ml solution of potassium oxonate (PO, 250 mg/kg) and adenine (75 mg/kg) by gavage, and NC group mice were given the equal volume of 0.5% CMC-Na solution for 14 days. Four hours after the treatment of PO and adenine, mice in LPP group were orally administered with 1 × 10^9^ CFU LPP in 0.3 ml PBS and mice in NC and HUA groups were given the equal volume of sterile PBS. After 14-day of daily administration of LPP or PBS, mice were sacrificed and body weight was recorded. Blood was collected from the eye vein. To acquire the serum, the collected blood samples were left undisturbed for 30 min then centrifuged at 5000 x*g* for 20 min. The kidney, liver, ileum, colon, and cecal content were also collected. All samples were stored at −80°C until analysis, except for the samples used for histopathology and RNA sequencing, which were kept in 4% paraformaldehyde and RNAlater (Thermo Fisher Scientific, Waltham, MA, United States) respectively.

### 2.5. Detection of biochemical indicators of HUA

SUA, serum creatinine (CRE), and blood urea nitrogen (BUN) concentrations were determined with commercial kits from the Jiancheng Biotech (Nanjing, China), and Xanthine oxidase (XOD) and adenosine deaminase (ADA) activities in livers were determined with commercial kits form the Jiancheng Biotech (Nanjing, China), following the manufacturer’s instructions.

### 2.6. Histopathological examination

Kidney tissues were collected and fixed in 4% paraformaldehyde solution for 24 h and the fixed tissues were embedded in paraffin, sliced into 4 μm sections, then stained with Hematoxylin and Eosin by Wuhan Servicebio Technology Co., Ltd. The HE sections were pictured and analyzed by CaseViewer software.

### 2.7. Transcriptome analysis

Transcriptome analysis for kidney and liver samples was performed by the Beijing Genomics Institute Co., Ltd., (Beijing, China). Briefly, the Total RNA of kidney and liver tissues was extracted using Trizol according to the manufacturer’s instruction. The qualified RNA sample, determined by Fragment Analyzer, was constructed data library. After enrichment and specific amplification of mRNA, the single-strand circular DNA library obtained was sequenced on the BGISEQ-500 platform by the Beijing Genomics Institute Co., Ltd. The clean data obtained from raw data by removing adapter-containing and low-quality reads were mapped to the reference genome using HISAT software for the second quality control of alignment. The matched clean data were normalized to FPKM by RSEM software with a threshold at |log2FC| ≥ 1, *p* ≤ 0.01. KEGG pathway enrichment of differential genes was performed on the Dr. Tom analysis platform of Beijing Genomics Institute Co., Ltd. The heatmap of differential genes was exerted by online tools^3^.

### 2.8. Quantitative real-time PCR analysis

RNA of kidney, colon and ileum tissue was extracted using Trizol and was reverse-transcribed using a Primescript RT master mix kit (Takara, Shiga, Japan). The cDNA samples were amplified in duplicate. Quantitative real-time PCR (qPCR) was conducted on Bioer Fast 9,600 (Bioer Technology, Hangzhou, China) with TB Green Premix Ex Taq. qPCR was run under the following conditions: pre-denaturation at 95°C for 30 s, denaturation at 95°C for 5 s, and annealing at 60°C for 30 s followed by 40 cycles. The primer sequences of genes were designed using BLAST and listed as supporting information in Supplementary materials (Supplementary Table S1). Gene expression was normalized with β-actin, and 2 − ∆∆Ct was used to calculate the result.

### 2.9. Western blot analysis

Colon and kidney tissues were homogenized in iced RIPA (Solarbio Science & Technology, Beijing, China) and proteins were extracted following the manufacturer’s instructions. The concentration of proteins was quantified using BCA protein assay kit (Thermo Scientific, MA, United States). Twenty-five micrograms of denatured protein were separated by 12% SDS-PAGE gel and transferred to an Immobilon-P Transfer Membrane (Millipore, Burlington, MA, United States). The membranes were blocked with 5% non-fat milk and incubated with primary antibodies (1:1000) overnight at 4°C. Then the membranes were incubated with horseradish peroxidase- (HRP-) conjugated secondary antibodies (1,5,000). All primary antibodies were purchased from Abcam plc., (Cambridge, United Kingdom), and HRP-secondary antibodies were purchased from Lablead Biotech Co., Ltd. (Beijing, China). The bands were visualized using ECL with Amersham Imager 680R (General Electric, Connecticut, United States). The grey values of the bands were calculated and normalized to those of β-actin using Image J software.

### 2.10. 16S rRNA sequencing and data analysis of cecal content

At the end of the experiment, fresh cecal content was collected and immediately stored at −80°C. 16S rRNA sequencing and data analysis were performed by the Beijing Genomics Institute Co., Ltd., (hBeijing, China). Briefly, V3 and V4 hypervariable regions of 16S rRNA were selected and amplified using the primer pairs: Forward primer (5′-CCTACGGGNGGCWGCAG-3′) and Reverse primer (5′-GACTACHVGGGTATCTAATCC-3′), and the amplicons and quality control of the raw data were conducted on the Illumina Hiseq 2,500 platform. The consensus sequence was generated by FLASH. The high-quality paired-end reads were combined into tags based on overlaps, then tags were clustered into Operational Taxonomic Unit (OTU) by scripts of software USEARCH with a 97% threshold. The α adversity and principal coordinates analysis (PCoA) was calculated and visualized by R software. Abundance prediction results of KEGG functions in bacterial communities were obtained by Phylogenetic Investigation of Communities by Reconstruction of Unobserved States (PICRUST2) (27). The functions are named by KO ID, which represents the specific functional genes, and then the information on 3 levels of metabolic pathways is obtained from the KEGG database. The abundance table of each level is obtained separately.

### 2.11. FITC-dextran permeability assay

The intestinal permeability was assessed using FITC-dextran (FD4, Sigma) method. Briefly, after being deprived of water and food for 12 h, mice were subjected to FITC-dextran gavage at the dose of 400 mg/kg body weight *via* a gastric gavage tube. Four hours later, blood was collected from inner canthus, and serum was collected by centrifugation (4°C 5000 × g 5 min). The FD-4 level was detected with multifunctional enzyme marker at 485 nm (excitation) and 528 nm (emission).

### 2.12. Statistical analysis

Statistical analysis was performed with GraphPad Prism (v9.1.0). The normal distribution data, which passed Levene test, were analyzed using one-way analysis of variance (ANOVA) followed by multiple comparisons (Tukey’s test) as post-hoc analysis to determine whether there was a statistical difference between every two sets of data. Results are presented as means ± standard deviation (SD). The nonparametric Kruska Wallis rank-sum test was used followed by Duncan’s test for multiple comparisons for alpha diversity analysis of the gut microbiota. *p* < 0.05 was considered statistically significant.

## 3. Results

### 3.1. Effect of LPP on body weight and biochemical indicators related to HUA

**Figure 1:**
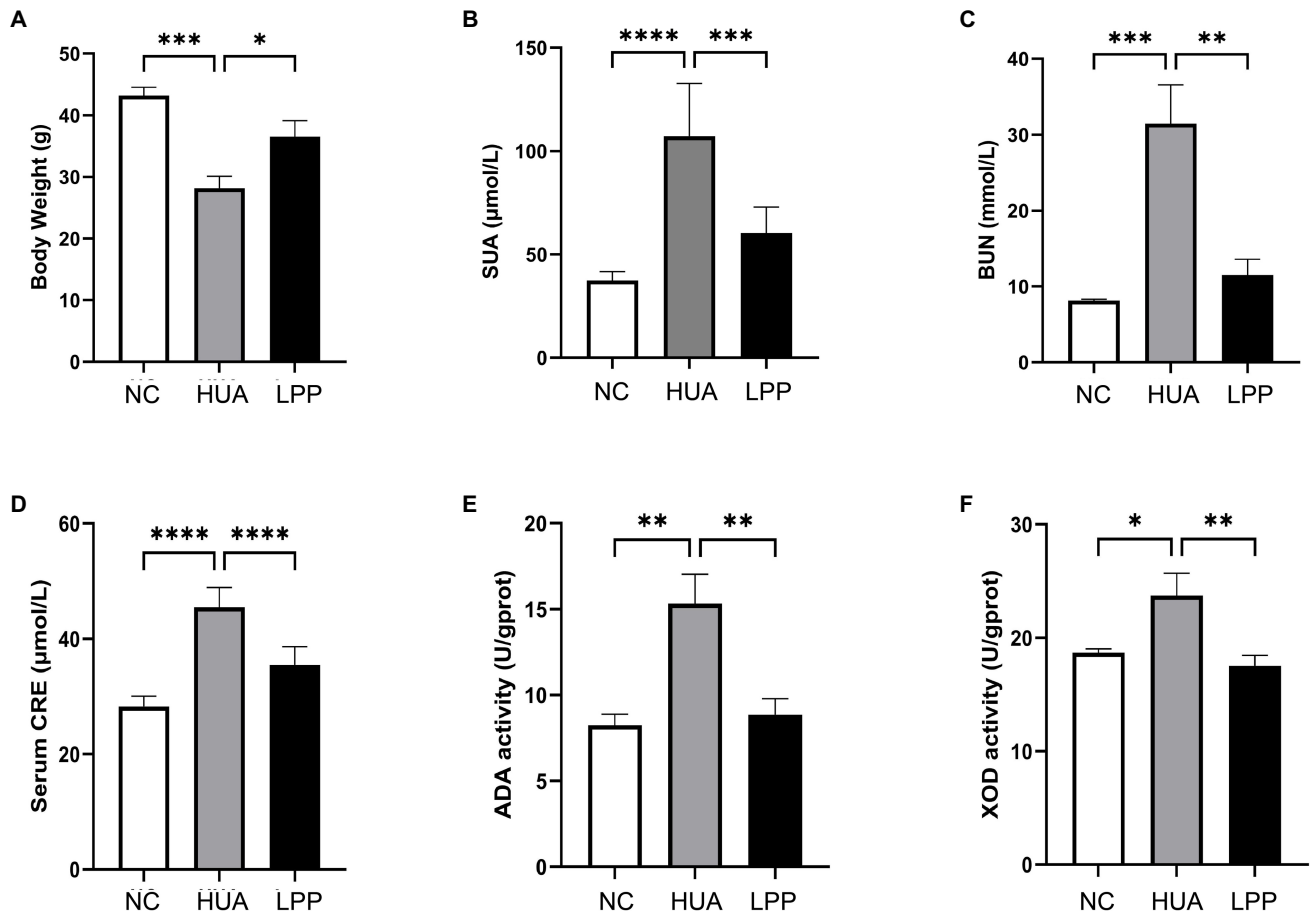
Effect of LPP on body weight **(A)**, levels of SUA **(B)**, BUN **(C)**, and CRE **(D)**, and activities of ADA **(E)** and XOD **(F)** in liver. NC, negtive control group; HUA, hyperuricemic group; LPP, hyperuricemic mice treated with LPP orally. Bars are means ± SD (*n* = 6/group), *p* < 0.05 was set as the threshold for significance by one-way ANOVA followed by *post hoc* comparisons using Tukey’s test for multiple groups’ comparisons, *p < 0.05; **p < 0.01; ***p < 0.001; ****p < 0.0001.

We first examined the body weight of the different groups of mice (Figure 1A), as body weight is a visual indicator of the overall health status of mice. Compared with NC mice, the HUA mice had significantly lower body weight, indicating their worse health condition. LPP treatment prevented the weight loss observed in HUA mice, suggesting the beneficial effect of LPP on the overall health status of mice. Then we measured the SUA concentration in mice as well as the levels of some important indicators related to the development of HUA. The concentration of SUA was significantly increased in the HUA mice compared with NC mice (107.19 ± 25.48 μmol/l vs. 37.375 ± 4.26 μmol/l, *p* < 0.0001), which confirmed the success of establishing a HUA mouse model. Orally administration of LPP significantly decreased the SUA level compared with the untreated HUA mice (60.33 ± 12.60 μmol/l vs. 107.19 ± 25.48 μmol/l, *p* < 0.001), which thus confirmed the SUA-lowering ability of LPP (Figure 1B). BUN and serum CRE are important indicators of renal injury, and dysfunction of the kidney occurs commonly in HUA mice. We observed that HUA mice had significantly higher levels of BUN and CRE compared with NC mice, and oral administration of LPP effectively reversed the elevated levels of BUN and CRE caused by HUA (Figures 1C,D). These results indicate that LPP is protective against renal injure. Since UA is mainly synthesized in the liver, we next examined the activity of ADA and XOD in liver, the two essential enzymes for the synthesis of uric acid. We found that ADA and XOD activities were remarkably elevated in the HUA mice compared to the NC mice, and the enhancements of both enzymes were restored by LPP intervention (Figures 1E,F). All these results indicated that oral administration of LPP could effectively lower the SUA level and relieve the symptoms of HUA.

### 3.2. Protective effect of LPP on HUA-induced renal inflammation and pathology

**Figure 2:**
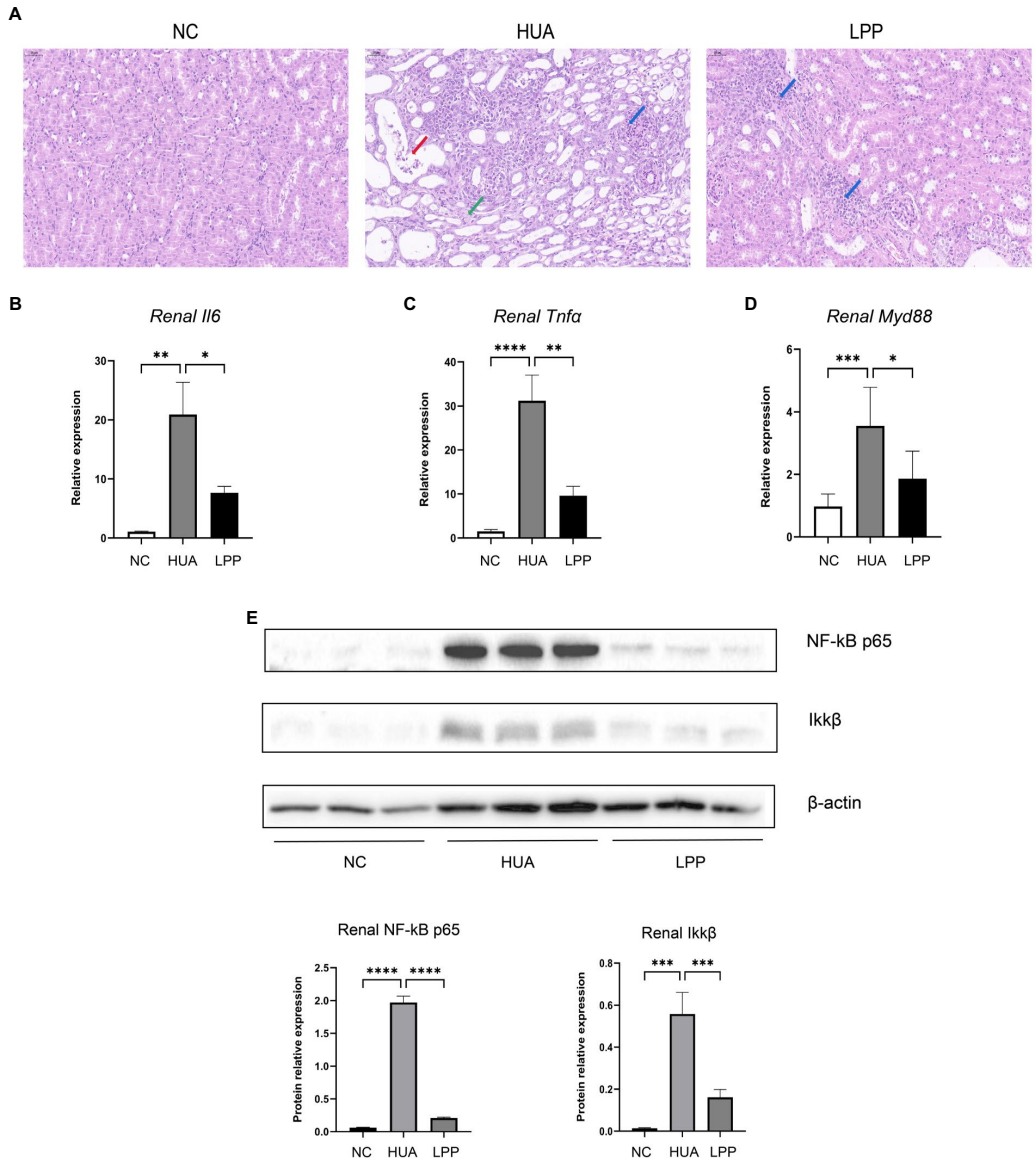
Protective effect of LPP on HUA-induced renal inflammation and pathology. **(A)** H&E stain of renal tissue (200×), red arrow: necrotic cell fragments; green arrow: interstitial fibrosis; blue arrow: inflammatory cell infiltration. **(B–D)** Quantitative PCR results of *Il-6* **(B)**, *Tnfa* **(C)**, and *Myd88* **(D)** relative expression standardized by β-actin (*n* = 6 per group). **(E)** Representative western blotting images of NF-kB p65 and Ikkβ in renal tissue. The protein expressions were quantitated by Image J software and normalized by β-actin (*n* = 3 per group). Bars are means ± SD, *p* < 0.05 was set as the threshold for significance by one-way ANOVA followed by *post hoc* comparisons using Tukey’s test for multiple groups’ comparisons, *p < 0.05; **p < 0.01; ***p < 0.001; ****p < 0.0001.

We next examined the inflammatory damage of kidney caused by HUA in different groups. Histopathological examination showed necrotic cell fragments in the lumen of both renal tubules and collecting ducts, and multiple interstitial cell fibrosis of the tissue together with inflammatory cell infiltration in the kidney of HUA mice (Figure 2A). While LPP treatment reversed the damages induced by HUA, inflammatory cell infiltration could still be found in kidney tissues. We then examined the levels of inflammation-related cytokines and proteins. Quantitative PCR results showed that kidney expression of *Il6, Tnfa,* and *Myd88* was significantly increased in HUA mice compared with NC mice, which was reversed by LPP treatment (Figures 2B–D). These results suggest that NF-kB pathway may be involved in renal inflammation in HUA mice and LPP administration may protect the kidney from the damage by inhibiting NF-kB pathway. To confirm the inhibitory effect of LPP on NF-kB pathway at protein level, protein levels of NF-kB p65 and Ikkβ, the most important proteins in NF-kB pathway, were examined by Western blotting. The results were consistent with those found at mRNA level (Figure 2E). Together, our data provided evidence supporting an anti-inflammatory role of LPP in alleviating HUA-related renal inflammation by downregulating NF-kB pathway.

### 3.3. *Lactiplantibacillus pentosus* P2020 altered expression of uric acid transport-related proteins in renal tissue

**Figure 3:**
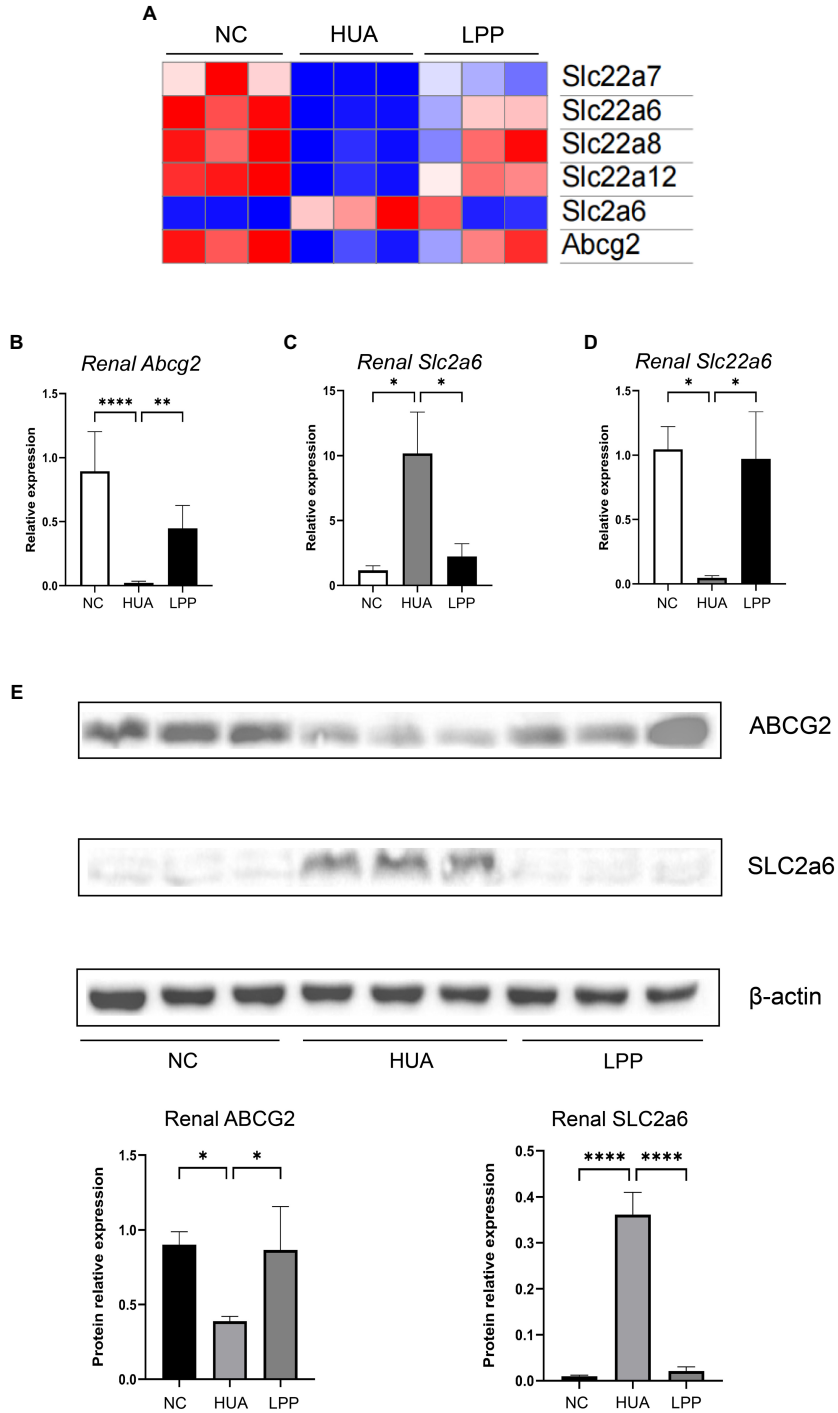
LPP altered the expression of uric acid transport-related proteins in renal tissue. **(A)** Heatmap of expression of UA transporters in renal measured by RNA-seq. (**B–D**) Relative expression of renal *Abcg2* **(B)**, *Slc2a6* **(C)** and *Slc22a6* **(D)** mRNA normalized with β-actin (*n* = 6 per group). **(E)** Representative western blotting image of ABCG2 and SLC2a6 in renal tissue. The protein expressions were quantitated by Image J software and normalized by β-actin (*n* = 3 per group). Bars are means ± SD, *p* < 0.05 was set as the threshold for significance by one-way ANOVA followed by *post hoc* comparisons using Tukey’s test for multiple groups’ comparisons, *p < 0.05; **p < 0.01; ***p < 0.001; ****p < 0.0001.

Several renal transporters are involved in the process of UA extraction, including both excretion and reabsorption of uric acid. It has been known that SLC2a6 (GLUT9) and SLC22a12 (URAT1) contribute to UA reabsorption, while ABCG2 and SLC22a6/7/8 (OAT1/2/3) are related to UA excretion. The results of RNA sequencing showed that expression of *Abcg2* and *Slc22a6/7/8* was significantly down-regulated and expression of *Slc2a6* was up-regulated in the HUA mice compared with the NC mice, which indicated less UA excreted into renal tubules and more UA reabsorbed into the blood in HUA mice compared with NC mice (Figure 3A). However, we also found that in the HUA group, *Slc22a12* expression was downgraded, implying that ABCG2 might have a stronger influence on UA reabsorption. We further observed that administration of LPP significantly reversed altered expression of *Abcg2*, *Slc22a6/7/8/12,* and *Slc2a6* caused by HUA, indicating an improvement of UA excretion in renal and UA reabsorption into blood mediated by LPP (Figure 3A). Selecting *Abcg2*, *Slc2a6,* and *Slc22a6* genes for quantitative PCR validation, we confirmed the results of RNA-sequencing (Figures 3B–D). Furthermore, we evaluated expression of the transporters at protein level by Western blotting and observed that the level of ABCG2 was dramatically decreased while the level of SLC2a6 was significantly increased in the renal tissue of HUA mice compared with NC mice. The LPP administration partially reversed these alterations observed in HUA mice, suggesting that oral administration of LPP might prevent HUA development by both promoting UA excretion *via* urine and reducing UA reabsorption into blood (Figure 3E).

### 3.4. Effect of LPP on intestinal barrier and transporters in the intestine

**Figure 4:**
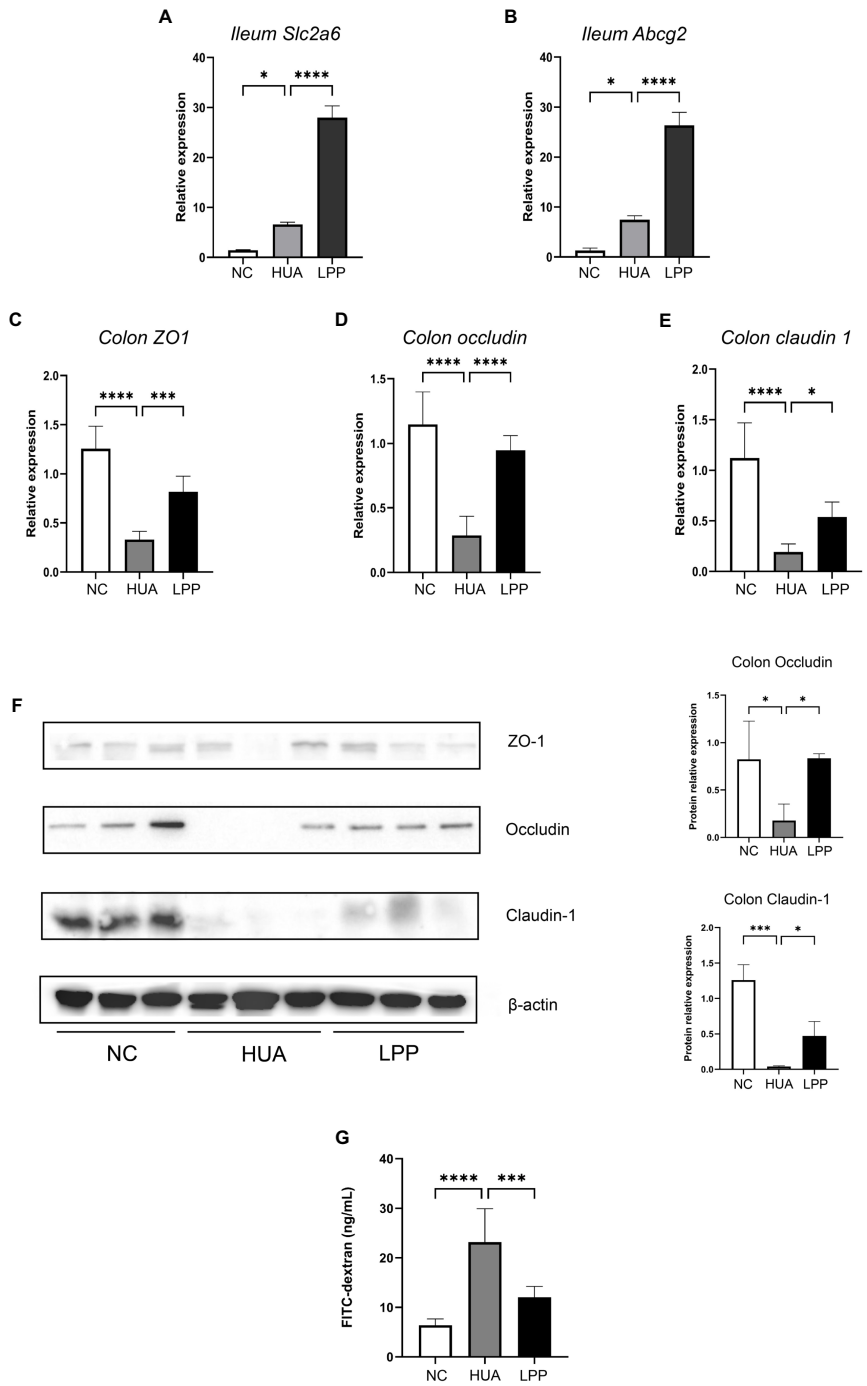
Effect of LPP on expression of tight junction protein and transporters in intestine. **(A,B)** Relative expression of *Slc2a6* and *Abcg2* mRNA in ileum tissue normalized by β-actin (*n* = 6 pre group). **(C–E)** Relative expression of TJ proteins (*ZO1, occludin, claudin1*) mRNA in colon tissue normalized by β-actin (*n* = 6 per group). **(F)** Representative western blotting images of ZO-1, Occludin and Claudin-1 in renal tissue. The protein expressions were quantitated by Image J software and normalized by β-actin (*n* = 3 per group). **(G)** FITC-dextran detected in serum of mice from NC, HUA and LPP groups after administration with oral gavage. Bars are means ± SD, *p* < 0.05 was set as the threshold for significance by one-way ANOVA followed by *post hoc* comparisons using Tukey’s test for multiple groups’ comparisons, *p < 0.05; **p < 0.01; ***p < 0.001; ****p < 0.0001.

About one-third of uric acid excretion takes place in the intestine, and illeum is the main site where intestinal excretion of uric acid occurs (28). It has been shown that uric acid transporters synthesized by intestinal microbes appear to integrate into the intestinal wall to regulate uric acid excretion (29). Therefore, we examined expression of two important UA transporters, ABCG2 and SLC2a6, to determine whether LPP affects the UA transporters in the intestine. The qPCR and Western blot results showed that expression of ABCG2 and SLC2a6 was enhanced in the ileum of HUA mice in contrast to NC mice. We also observed that administration of LPP further increased the levels of ABCG2 and SLC2a6 compared with HUA mice, suggesting that administration of LPP might reduce the serum level of UA *via* influencing expression of UA transporters in the ileum (Figures 4A,B).

Since it has been reported that destruction of the intestine barrier correlates with the hyperuricemic condition (30), we wondered whether LPP could ameliorate the impaired intestinal barrier caused by HUA. The quantitative PCR and Western blot (Figures 4C–F) results showed that expression of tight junction (TJ) proteins Occludin and Claudin-1 in colon tissue was decreased under the high SUA condition, which reflects the disruption of intestinal barrier function due to high SUA level. However, we did not observe a significant decrease in ZO-1 protein expression (statistical data not shown), although a decrease was observed at its mRNA level. The finding that LPP treatment partially reversed the reduction of TJ protein expression provides evidence supporting the idea that oral intake of probiotics can improve intestinal barrier function. To further determine whether LPP gavage could improve the disturbed intestinal barrier induced by HUA, intestinal permeability was monitored uisng FITC-dextran (4 kD) method *in vivo*. As shown in Figure 4G, FITC-dextran concentration in serum was significantly reduced in LPP group, which confirmed the ability of LPP to attenuate the damage of intestinal barrier. With all these results together, we conclude that oral administration of LPP may have a protective effect on HUA development and the mechanisms for this effect involve increased expression of tight junction proteins and transporter proteins in the intestine.

### 3.5. Effect of LPP on gut microbiota in HUA mice

**Figure 5:**
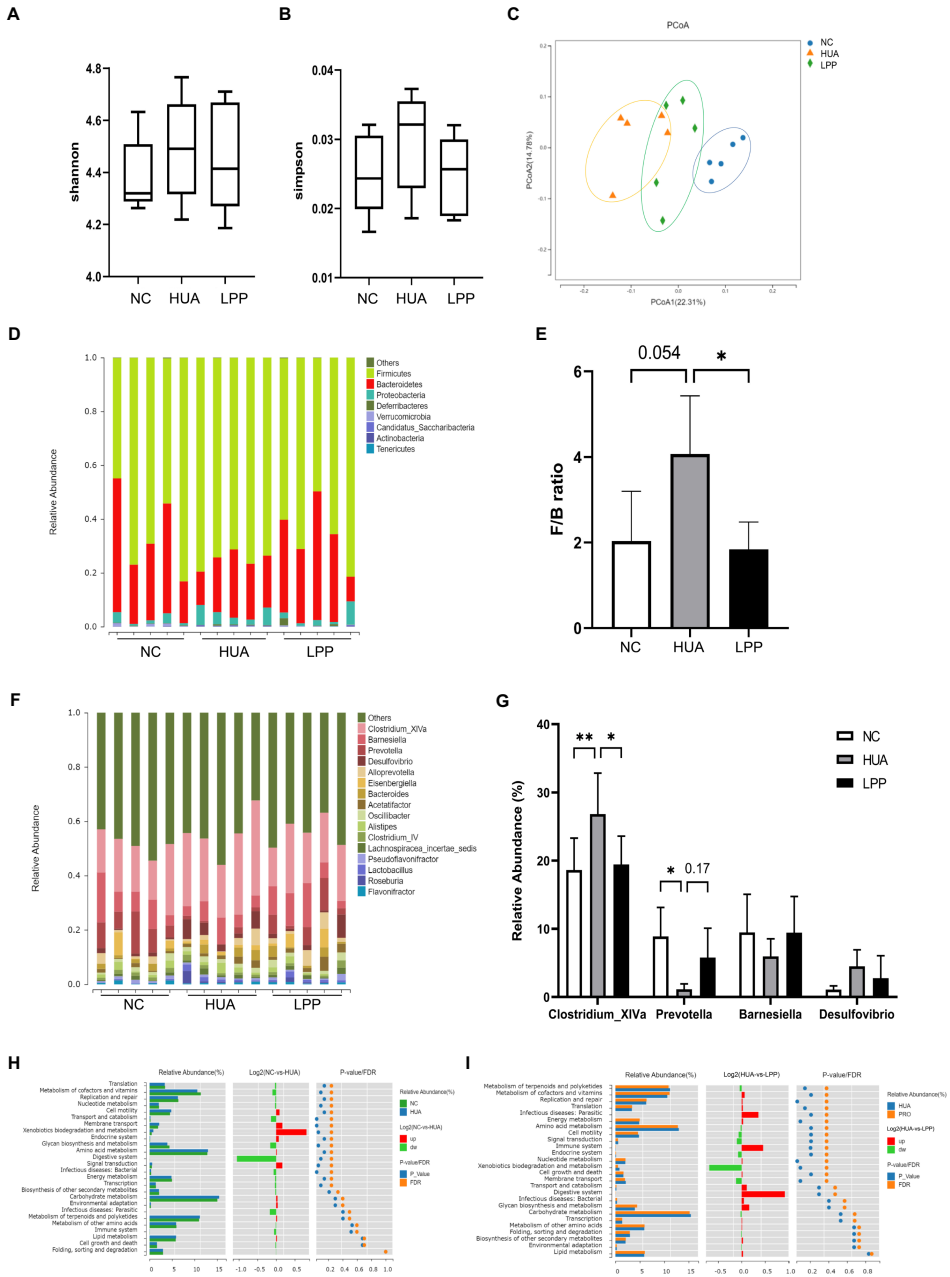
LPP restored alteration in gut microbiota of HUA mice. **(A,B)** Shannon Index and Simpson Index indicated α diversity in different groups. **(C)** Principal coordinate analysis (PCoA) plot based on unweighted UniFrac distance. **(D)** Comparison of phylum relative abundance in different groups. **(E)** The ratio of relative abundance of Firmicutes and Bacteroidetes (F/B ratio). **(F)** Comparison of genus relative abundance in different groups. **(G)** Relative abundance of the key differential genus in different groups. **(H,I)** Predicted microbial function comparisons. Gene functional categories were from level 2 of KEGG pathways. FDR, false discovery rate. **(H)** Comparing microbial function between the NC mice and HUA mice, the enriched functions in the NC mice were presented with green color and the enriched functions in the HUA mice were presented with blue color. **(I)** Comparing microbial function between the HUA mice and LPP-treated mice, the enriched functions in the HUA mice were presented with blue color and the enriched functions in the LPP-treated mice were presented with orange color. The nonparametric Kruska Wallis rank-sum test was used followed by Duncan’s test for multiple comparisons for alpha diversity analysis of the gut microbiota. Other data were analyzed by one-way ANOVA followed by *post hoc* comparisons using Tukey’s test for multiple groups’ comparisons, *p < 0.05; **p < 0.01; ***p < 0.001; ****p < 0.0001.

Since both HUA status and oral administration of probiotics may change the composition of intestinal flora, we investigated how LPP impacts the gut microbiota of HUA mice using 16S rRNA sequencing. The Shannon Diversity Index and the Simpson Diversity Index were evaluated to determine the alpha diversity of gut microbiota. As shown in Figures 5A,B, there was no significant difference in the alpha diversity of gut flora among the three groups. To analyze the beta diversity, the distance for each sample was calculated using the presence or absence of microbial flora (unweighted) and displayed in the coordinate space. The PCoA analysis showed a significant difference in beta diversity of gut microbiota between HUA and NC mice, and LPP treatment partially reversed this alteration in HUA mice (Figure 5C). Furthermore, at the phylum level, *Bacteroidetes, Firmicutes, Proteobacteria, Verrucomicrobiota*, and *Deferribacteres* constituted the main microbiota in all three groups with *Bacteroidetes* and *Firmicutes* being dominant (Figure 5D). The *Firmicutes/Bacteroidetes* ratio (F/B ratio) in HUA mice was significantly higher than that in NC mice, while the F/B ratio was reversed after LPP treatment (Figure 5E). At the genus level, the gut microbiota was mainly composed of *Clostridium XlVa*, *Barnesiella*, *Prevotella*, *Eisenbergiella*, *Alloprevotella*, *Alistipes*, *Bacteroides*, *Oscillibacter* and *Clostridium IV* (Figure 5F). The relative abundance of *Clostridium XlVa* was significantly higher and *Prevotella* was lower in HUA group than NC group (*Clostridium XlVa:* 26.97% in HUA vs. 18.67% in NC and *Prevotella:* 1.15% in HUA vs. 8.79% in NC; Figure 5F). Administration of LPP resulted in an increased abundance of *Prevotella* (5.75% in LPP vs. 1.15% in HUA) and decreased abundance of *Clostridium XlVa* (19.54% in LPP vs. 26.97% in HUA), suggesting that LPP may partially reverse HUA-induced alteration in gut microbiota (Figure 5G). Since alterations in bacterial taxa may also potentially affect the metabolic functions of the gut microbiota, we analyzed and compared the metabolic functions of the gut microbiota among different groups. As shown in Figure 5H, KEGG pathways in level 2 were different between NC and HUA groups. Compared with NC group, upregulated pathways in HUA mice were mainly related to xenobiotics biodegradation and metabolism, membrane transport, and cell motility. We noticed that these up-regulated pathways by HUA were downregulated by LPP treatment (Figure 5I). Together, these results indicate that treatment of LPP may not only attenuate HUA symptoms but also restore the composition and the function of gut microbiota toward the microbiota seen in normal mice.

### 3.6. LPP exerted anti-inflammatory potential mechanism underlying UA-induced inflammation and the protective effect of LPP transcriptome

**Figure 6:**
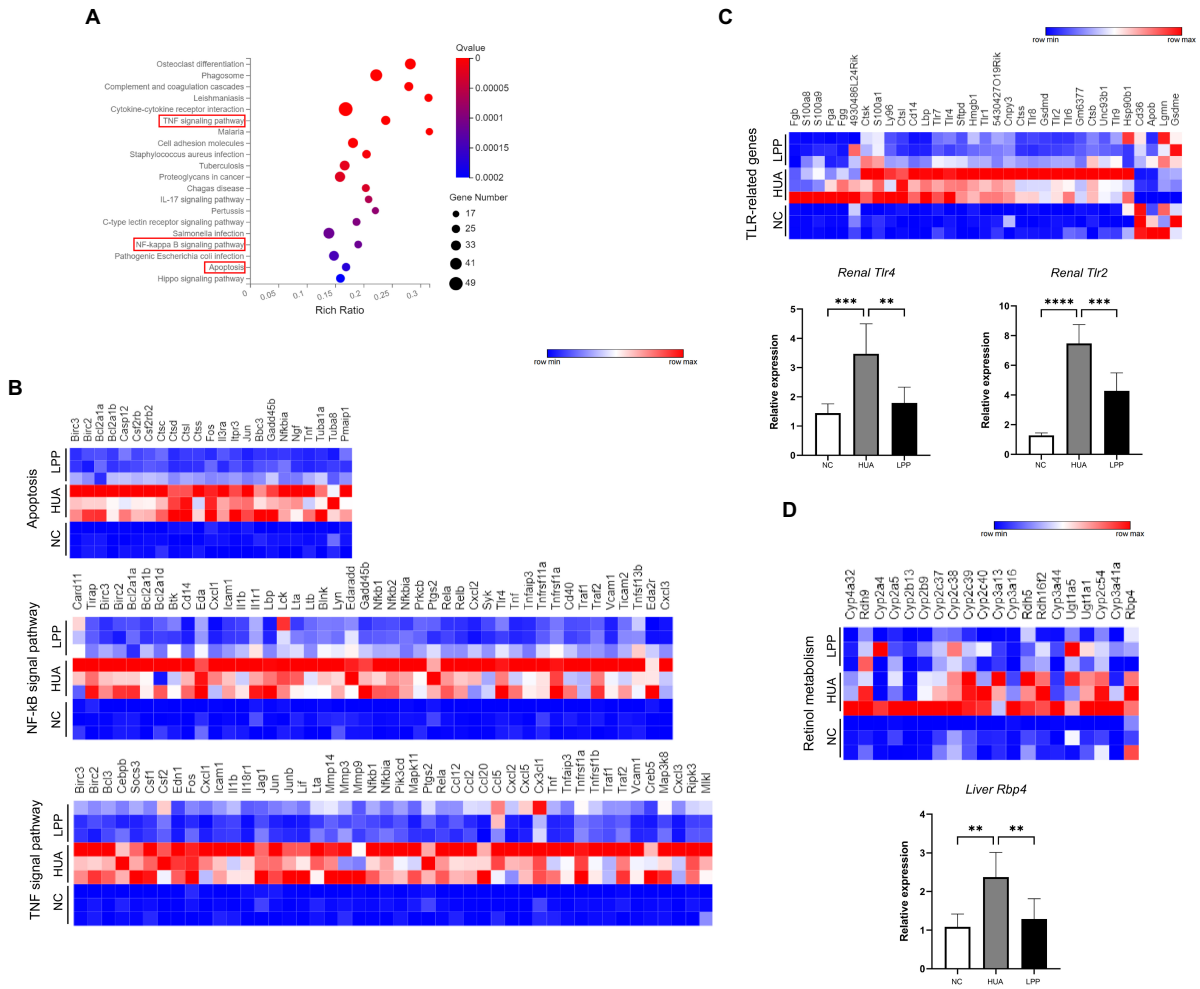
Transcriptome data revealed the potential mechanisms for UA-induced renal inflammation and LPP protective effect. **(A)** Bubble diagram of the top 20 KEGG pathways downregulated in the LPP group compared with the HUA group in renal tissue. **(B)** Heatmap depiction of the expression of apoptosis, TNF, and NF-kB signal pathway in renal tissue from different groups. **(C)** Heatmap depiction of the expression of TLR-related genes and qPCR results of *Tlr2/4* in renal tissue from different groups. **(D)** Heatmap depiction of retinol metabolism signal pathway and qPCR results of *Rbp4* in liver tissue from different groups. Bars are means ± SD, *p* < 0.05 was set as the threshold for significance by one-way ANOVA followed by *post hoc* comparisons using Tukey’s test for multiple groups’ comparisons, *p < 0.05; **p < 0.01; ***p < 0.001; ****p < 0.0001.

To explore the mechanism of how oral administration of LPP protects the renal injury caused by HUA, transcriptome analysis for the kidneys from all groups was conducted. The volcano diagram showed that 2,535 differential expressed genes (DEGs) including1372-upregulated and 1,163-downregulated genes between HUA and LPP groups were identified (Supplementary Figure S1). The bubble diagram showed the top 20 KEGG pathways upregulated in the LPP group compared with the HUA group, in which TNF, NF-kB, and MAPK signaling pathways were closely associated with tissue inflammation and necrosis (Figure 6A). The heatmap of gene expression enriched in MAPK, TNF and NF-kB signal pathways showed that expression of genes such as *Tnf, Fos, Jun, Junb, Ccl2/5/12/20, Cx3cl1* was dramatically upregulated in HUA mice. Administration of LPP could significantly downregulate these genes involved in MAPK, TNF, and NF-kB signal pathways, suggesting that LPP could attenuate HUA-induced inflammatory pathological processes in renal tissues (Figure 6B). Literature has demonstrated that UA can activate inflammatory pathways by binding Toll-like receptor (TLR) 2 and TLR4 (31). To understand whether LPP administration affects expression of TLR-related genes, we examined expression of *Tlr4*, *Lbp* and *Cd14*, and *Tlr2* and found higher expression of these gene in the kidney of HUA mice compared with NC mice and a reversal of these changes by LPP administration. The results of q-PCR confirmed the transcriptomic results (Figure 6C). Besides, expression levels of other TLRs (*Tlr1/6/7/8/9*) were also changed in HUA mice, which indicates that other TLRs other than TLR4 and TLR2 may also be involved in kidney injury caused by UA.

The liver is an important organ for UA metabolism, and liver transcriptome results may reveal some clues regarding how LPP affects SUA levels. We identified 1,200 DEGs (868 upregulated, 332 downregulated) between HUA and LPP groups (Supplementary Figure S2). A bubble diagram of the top 20 KEGG pathways downregulated in the LPP group indicated that retinol metabolism may be affected by LPP intake (Supplementary Figure S2). Many clinical and animal studies have shown that development of HUA is associated with upregulation of the retinol metabolic pathway or high dose of vitamin A (one of the products of retinol metabolism) intake (32–34). Retinol dehydrogenase (RDH) is the key enzyme for vitamin A production. Consistent with other studies, our transcriptomic data showed that the expressions of *Rdh5*, *Rdh9,* and *Rdh16f2* were significantly increased in HUA mice and LPP intake could partially reverse the changes in these genes (Figure 6D). Retinol-binding protein 4 (RBP4) plays a key role in retinol and vitamin A metabolism (35). RBP4 belongs to the apolipoprotein family and transports retinol from the liver to peripheral tissues as the retinol-specific carrier in the blood. Therefore, we verified the transcriptome data on *Rbp4* by q-PCR and confirmed that *Rbp4* mRNA level was significantly elevated under HUA status, and this upregulation was suppressed by oral LPP administration.

## 4. Discussion

Primary HUA induced by genetic factors is uncommon in the population, while secondary HUA driven by a high purine diet is mainstream (36). The use of UA-lowering drugs is not the first choice for patients with asymptomatic HUA (37). However, patient compliance and outcomes are often unsatisfactory with simple diet control therapy. Thus, probiotics administration is an alternative adjunctive treatment of HUA. Among the probiotic strains, *Lactobacillus* showed diverse biological functions (38, 39). Only a few *Lactobacillus* strains have been reported to alleviate HUA in the *in vivo* studies. In this study, we isolated and evaluated a candidate probiotic strain LPP for its UA-lowering function. Our results showed that LPP could significantly lower SUA levels and relieve symptoms of HUA.

The liver is the main site of uric acid synthesis, and XOD and ADA play a key role in this process (40). Our results showed that activity of ADA and XOD in the liver of HUA mice was significantly higher than that in NC mice, and oral administration of LPP could suppress the activity of ADA and XOD. Previous studies have demonstrated that anti-inflammation bioactivity and short-chain fatty acids (SCFAs) induced by probiotics may suppress XOD activity in the liver since the pro-inflammation factors such as LPS and IL-1β can increase XOD activity (22, 41, 42). In this study, we observed that oral administration of LPP displayed anti-inflammatory activity in the kidney, which may contribute to suppressing XOD activity in the liver through an unidentified mechanism. Although the importance of the liver for uric acid synthesis is well recognized, the transcriptomics of the liver has rarely been studied. To our surprise, the liver transcriptome revealed a possible relationship between the retinol metabolic pathway and HUA, suggesting that LPP may reduce SUA by affecting this pathway. Although the interaction between UA synthesis, retinol metabolism, and intestinal flora remains unclear, we speculate that influencing retinol metabolism by regulating gut microbiota may be a possible solution to prevent HUA.

Both crystallized and soluble UA can induce inflammation in multiple organs and tissues, and the kidney is one of the main organs affected during the long-term HUA state. The main objectives of the current clinical treatment of HUA are (1) to inhibit XOD activity, (2) to affect the transport of UA in the kidney to enhance UA excretion, and (3) to alkalize urine to improve UA excretion (43, 44). These drugs do not have an anti-inflammatory effect and cannot relieve the HUA-related inflammatory damage in the kidney. In this study, we identified the anti-inflammation effect of LPP on HUA-induced renal inflammatory lesions by inhibiting NF-kB pathway at both transcriptional and translational levels. Based on our result, LPP has the potential value as adjuvant medicine to alleviate renal injury caused by HUA. Additionally, our transcriptomic data suggest that UA-induced renal inflammation may be initiated by extensive activation of TLRs (*Tlr2/4/1/6/7/8/9*). NF-kB pathway is triggered by TLRs’ activation. It has been reported that secretions from some specific strains can suppress inflammatory reactions in a TLR4-dependent way (45, 46). Similarly, we speculate that certain small molecules produced by LPP may enter the circulation from the intestinal lumen and inhibit inflammatory pathways by interacting with various TLRs, resulting in inhibition of HUA-induced renal inflammation. However, the underlying mechanism as to how LPP interacts with TLRs remains to be elucidated.

About two-thirds of uric acid excretion is accomplished through the kidney, and members of solute carrier (SLC) family and ATP-binding cassette (ABC) superfamily play a significant role in this process (36). Several probiotics and prebiotics have been reported to promote uric acid excretion in animal studies by altering expression of uric acid transporters in the kidney, which in turn reduces SUA levels (22, 41, 47–49). For example, *Lacticaseibacillus paracasei* MJM60396 intake increased expression of OAT1 and OAT3 and decreased expression of URAT1 and GLUT9 in the HUA mouse model (47). To examine whether LPP would affect expression of uric acid transporters in the kidney, we found that administration of LPP significantly reversed the alteration in expression of genes related to UA excretion and reabsorption. Recently, studies on the interaction between gut microbiota and kidney (also known as “gut-kidney axis”) have intensified. Metabolites of intestinal flora are thought to enter the kidneys through circulation to perform their biological activities and to influence renal function (42). More studies are needed to specifically elucidate how the intestinal flora interacts with SLC and ABC family proteins.

Oral administration of probiotics can affect intestinal function directly. In addition to the excretion of UA *via* kidney, another important route of UA excretion is *via* the intestinal tract, which is responsible for about one-third of the total UA elimination. ABCG2 and SLC2a6 expressed in the intestinal epithelium translocate uric acid from the blood to the intestinal tract. When the renal excretion pathway of UA is disturbed, UA is compensated for by excretion from the intestine, resulting in elevated levels of uric acid in the patient’s stool (50, 51). As we expected, the levels of ABCG2 and SLC2a6 were both upregulated in the ileum tissue of mice in the HUA group. However, the expression of these two transporters elevated even higher after treatment with LPP. We speculate that oral intake of probiotics could enhance the intestinal excretion of uric acid by upregulating the expression of ABCG2 and SLC2a6, thereby reducing SUA concentration. Intestinal barrier function is related to systemic inflammation. A weakened intestinal barrier, also known as “gut leak,” can increase serum levels of pro-inflammatory substances such as lipopolysaccharides (LPS), which in turn can trigger a systemic inflammatory response (52, 53). Many prebiotics or probiotics have been reported to enhance intestinal barrier function. We observed impaired intestinal barrier function in HUA mice, which is consistent with some previous findings (30). The recovery of intestinal wall function may decrease serum LPS concentrations, lessen systemic inflammation, reduce XOD activity, and inhibit uric acid synthesis as previously described (30, 54, 55).

Similar to many metabolic diseases, HUA can also alter the intestinal microbiota. We found altered composition in intestinal flora of HUA mice at the phylum and genus levels, as evidenced by an increase in F/B ratio, the relative abundance of *Clostridium XIVa*, and a decrease in *Prevotella*. *Clostridium XIVa,* a butyrate-producing genus, has been reported to increase the number of Treg cells and ameliorate the symptoms of IBD (56). Nevertheless, some researchers reported that *Clostridium XIVa* contributed to the discrimination of obese patients with high UA, serum lipid, or blood pressure (57). *Prevotella* spp. is a member of the most dominant genera in the oral cavity. However, the function of *Prevotella* spp. and its contribution to host–microbiome crosstalk remains unclear (58), and conclusions regarding the relationship between *Prevotella* and human health are controversial. In a HUA rat model, genera level of *Prevotella* in fecal is decreased (14), suggesting that *Prevotella* may be negatively correlated with the systemic inflammatory response. In contrast, a study showed that increased *Prevotella* abundance is associated with augmented T helper type 17 (Th17) -mediated mucosal inflammation, which is in line with the marked capacity of *Prevotella* in driving Th17 immune responses *in vitro* (59). The interpretation and significance of these changes in *Clostridium XIVa* and *Prevotella* caused by LPP depend on further investigation.

## 5. Conclusion

In the present study, we isolated, identified, and evaluated a probiotic candidate strain LPP with HUA-lowering character. We found that LPP treatment suppressed inflammation in renal by inhibiting multiple pathways including NF-kB, TNF, MAPK signaling pathways. In addition, LPP treatment also regulated expression of UA transporters, protected the intestinal barrier, and restored the disrupted gut microbiota. This study has provided evidence supporting the application of LP*P* as an effective intervention to ameliorate HUA, which needs to be verified in humans in the future.

## Data availability statement

The datasets presented in this study can be found in online repositories. The names of the repository/repositories and accession number(s) can be found at: https://www.ncbi.nlm.nih.gov/genbank/, PRJNA896723, https://www.ncbi.nlm.nih.gov/, PRJNA897871, https://www.ncbi.nlm.nih.gov/, PRJNA898670, https://www.ncbi.nlm.nih.gov/, PRJNA899144.

## Ethics statement

The animal study was reviewed and approved by the Ethics Review Committee of the National Institute for Communicable Disease Control and Prevention at the Chinese Center for Disease Control and Prevention (Beijing, China).

## Author contributions

ZW and ZR conceptualized the experiments. ZW, LS, XL, YH, YX, YZ, JL, and ML conducted the experiments. ZW and YH analyzed the data. ZH and ZR wrote the paper. ZW, XL, and ZR discussed and revised the manuscript. All authors contributed to the article and approved the submitted version.

## Funding

This work was supported by grants from the National Natural Science Foundation of China (No. 81371761 to ZR), and the Ministry of Science and Technology of China (Grant No. 2018ZX10305409-003-001 to ZR).

## Conflict of interest

The authors declare that the research was conducted in the absence of any commercial or financial relationships that could be construed as a potential conflict of interest.

## Publisher’s note

All claims expressed in this article are solely those of the authors and do not necessarily represent those of their affiliated organizations, or those of the publisher, the editors and the reviewers. Any product that may be evaluated in this article, or claim that may be made by its manufacturer, is not guaranteed or endorsed by the publisher.

## Footnotes

^1^ https://ggdc.dsmz.de/

^2^ https://www.ezbiocloud.net/tools/ani

^3^ https://software.broadinstitute.org/morpheus/
