# Supplementary material for: Lactiplantibacillus pentosus P2020 protects the hyperuricemia and renal inflammation in mice
Source: Front Nutr. 2023 Feb 20;10:1094483. doi: 10.3389/fnut.2023.1094483 (PMC9987516; doi:10.3389/fnut.2023.1094483)
Supplement: Supplementary file 1 [file Data_Sheet_1.DOCX]

| Gene | Forward primer | Reverse primer |
| --- | --- | --- |
| *β-actin* | TGGATGACGATATCGCTGCG | AGGGTCAGGATACCTCTCTT |
| *Il6* | ACAAGTCGGAGGCTTAATTACACAT | TTGCCATTGCACAACTCTTTTC |
| *Tnfα* | CTGTAGCCCACGTCGTAGC | TTGAGATCCATGCCGTTG |
| *Myd88* | GGACAAACGCCGGAACTTTT | CATGCGGCGACACCTTTTCT |
| *Abcg2* | TTTGCCCTTCATTTCACGAGTGG | GCCGTTGTTGTTTCTCTGCG |
| *Slc2a6 (Glut9)* | CATGATCACAGAGCCACGCT | GAGCGAGAAGGACCATTTCTTTG |
| *Slc22a6 (Oat1)* | GCATAATACCGAGGGGCCATA | TCTGCCGAATCATTGTGGGG |
| *ZO1* | CCTAAGACCTGTAACCATCT | CTGATAGATATCTGGCTCCT |
| *occludin* | TCACTTCCTTCACTCTCTGTAG | CTGGCTTTTGATGCTCTTTGC |
| *claudin 1* | TGGGGCTGATCGCAATCTTT | CACTAATGTCGCCAGACCTGA |
| *Tlr4* | CCTGGACTTGGACCTCAG | GGACTGAAAGCTGCACATC |
| *Tlr2* | CTGATGCCAGGCTCCGTTC | TCCTCTGAGATTTGACCTCCTT |
| *Rbp4* | GCTCCTGCTGGGCAGACT | GTGGCGCTCATATGACCCTT |

Table S1. Primer Sequences


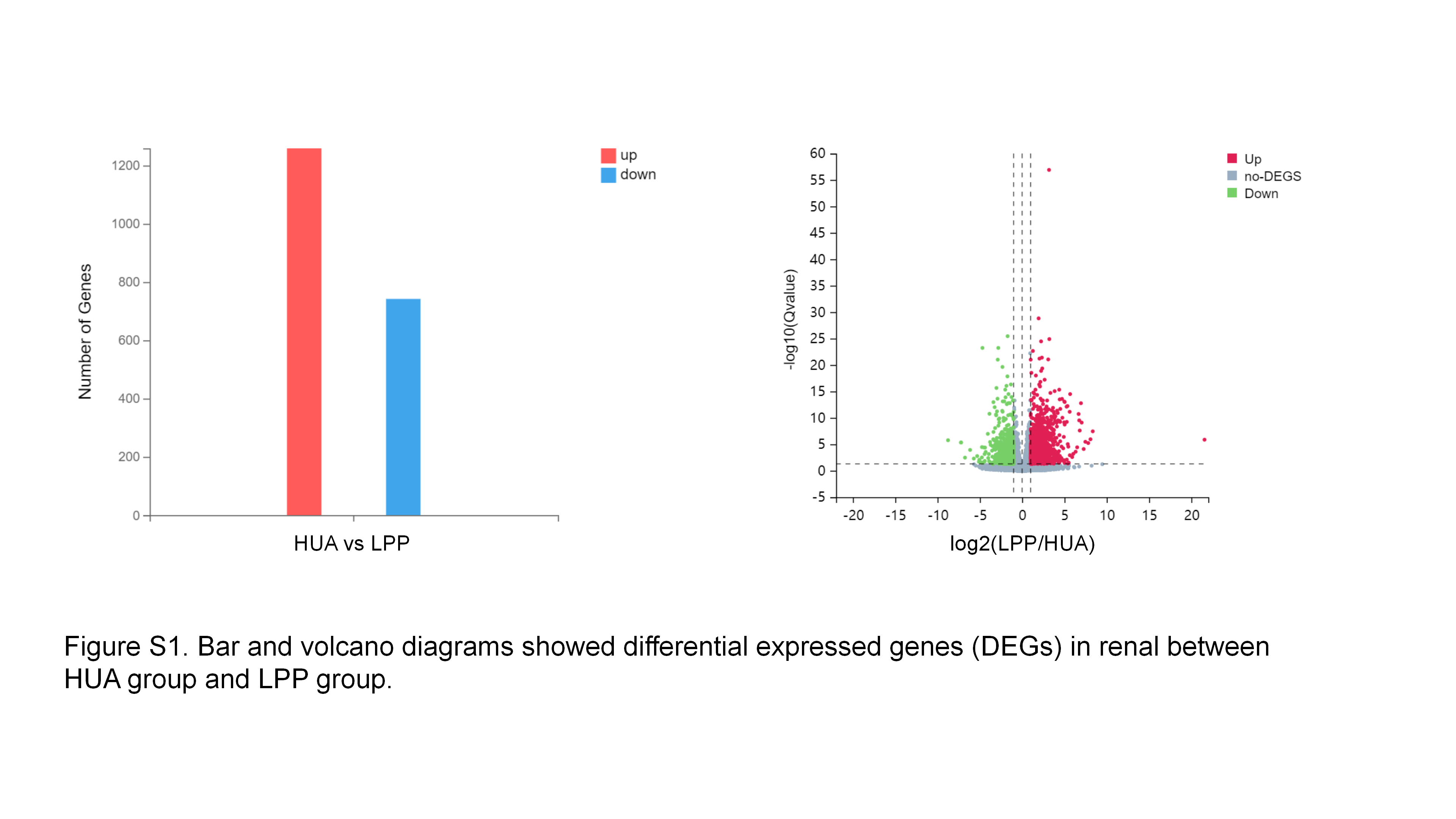


Figure S1. Bar and volcano diagrams showed differential expressed genes (DEGs) in renal between HUA group and LPP group.


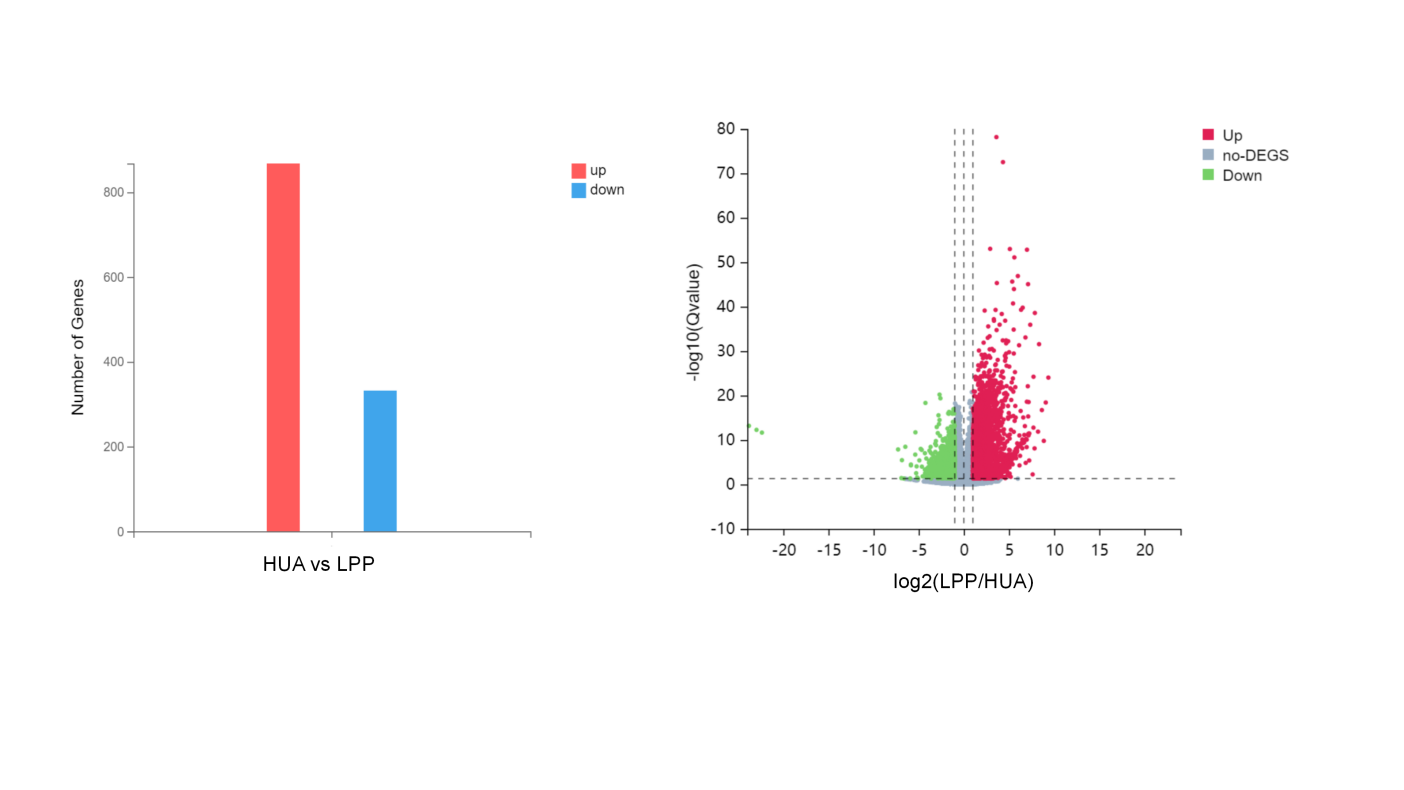


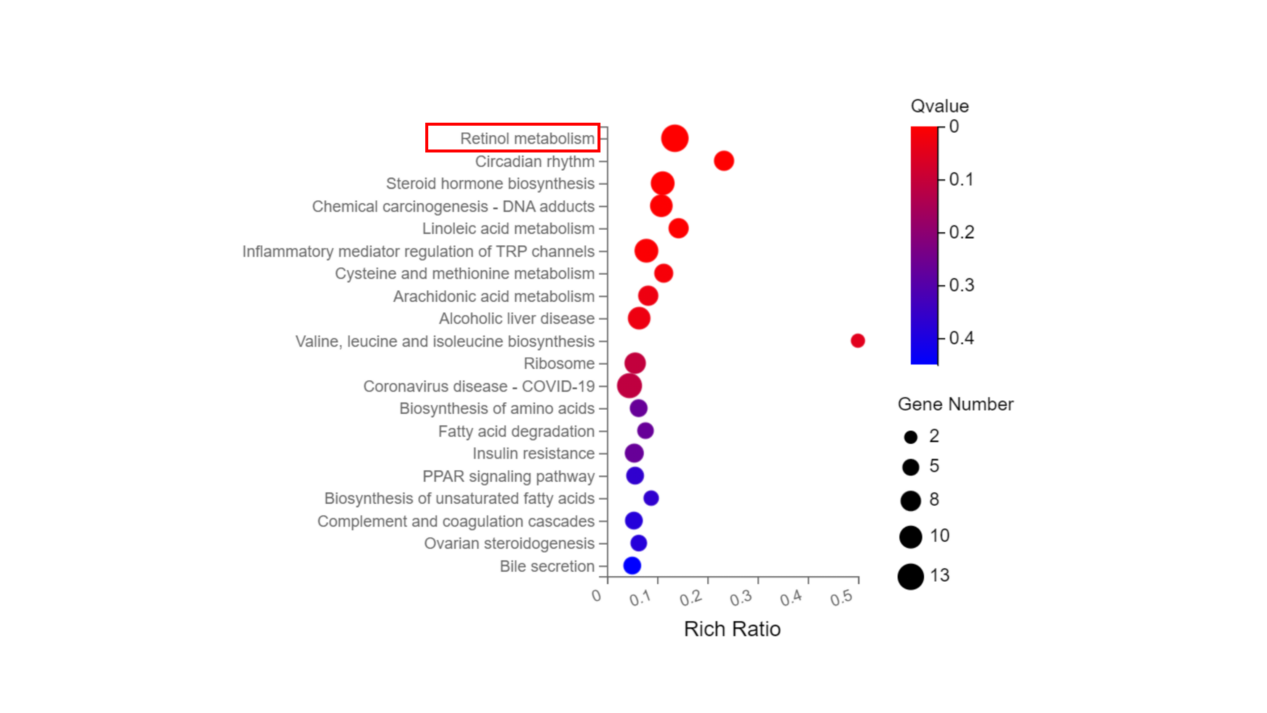


Figure S2. Bar and volcano diagrams showed differential expressed genes (DEGs) in liver between HUA group and LPP group. Bubble diagram showed the top 20 KEGG pathways in which genes downregulated in LPP group were enriched.
